# Gatekeeping in cancer clinical trials in Canada: The ethics of recruiting the “ideal” patient

**DOI:** 10.1002/cam4.3031

**Published:** 2020-04-20

**Authors:** Jennifer A. H. Bell, Mary T. Kelly, Karen Gelmon, Kim Chi, Anita Ho, Patricia Rodney, Lynda G. Balneaves

**Affiliations:** ^1^ University of Toronto Toronto ON Canada; ^2^ Princess Margaret Cancer Centre Toronto ON Canada; ^3^ University Health Network Toronto ON Canada; ^4^ University of British Columbia Vancouver BC Canada; ^5^ University of California Oakland CA USA; ^6^ Centre for Health Evaluation & Outcomes Sciences University of British Columbia Vancouver BC Canada; ^7^ University of Manitoba Winnipeg MB Canada

**Keywords:** clinical trials, oncology trials, research ethics, trial accrual, trial participants

## Abstract

**Background:**

Perspectives of clinical trial (CT) personnel on accrual to oncology CTs are relatively absent from the literature. This study explores CT personnel's experience recruiting patients to oncology CTs.

**Methods:**

A qualitative study design was utilized. In‐depth, individual interviews with 12 oncology CT personnel were conducted, including six CT nurses and six physician‐investigators. Interviews were digitally recorded and transcribed verbatim. Data were subjected to thematic and ethical analysis to identify key concepts and themes.

**Results:**

CT personnel reported considering two ethical commitments in CT recruitment: maintaining trial integrity and ensuring patient autonomy through obtaining informed consent. The process of gatekeeping emerged as a way to navigate these ethical commitments during CT accrual. Gatekeeping was influenced by: (a) perceptions of patients’ personal suitability for a trial, and (b) healthcare resources and infrastructure. CT personnel's discernment of personal suitability was influenced by patients’ cognitive and mental health status, language and cultural background, geographic location, family support, and disease status. Three structural factors impacted gatekeeping: complexity of CTs, consent process, and time limitations in the healthcare system. CT personnel experienced most factors as constraints to accrual and gaining patients’ informed consent.

**Conclusion:**

CT personnel discussed navigating ethical challenges in CT recruitment by offering enrollment to specific patient populations, exacerbating other ethical tensions. Systems‐level strategies are needed to address barriers to ethical CT recruitment. Future research should investigate the role of policies and/or tools (eg, decision aids) to support patients and CT personnel's discussions about CT participation, promote more ethical recruitment, and potentially increase accrual.

## Graphical abstract

The judgments of healthcare professionals may negatively influence equitable access and recruitment to clinical cancer trials, especially for patient populations marginalized by factors such as geography, poverty, mental health/addiction, immigration status, and language. Systems‐level strategies are needed to address the barriers that constrain ethical recruitment to clinical trials.

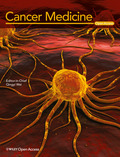

## 1 INTRODUCTION

Low accrual to cancer clinical trials (CTs) continues to challenge the translation of innovative research into clinical practice to improve patient outcomes. Research from the United States indicates that 80% of cancer trials are unable to reach accrual goals and only 3%‐5% of adult cancer patients participate in trials.^1^, ^2^ Unsuccessful accrual wastes valuable research funds and resources, and compromises investigators’ abilities to develop the evidence required to improve patient care.

There is a substantial body of literature on the barriers to patients’ participation in cancer CTs,^3^, ^4^, ^5^, ^6^, ^7^, ^8^, ^9^, ^10^, ^11^, ^12^, ^13^ with recent evidence confirming clinical and structural barriers as being most significant.^14^ However, perspectives of CT personnel are largely absent from this literature, with a few exceptions.^15^, ^16^, ^17^ One study examined physician‐investigators and research nurses’ perceived barriers to recruitment to radiation therapy CTs. An absence of formal mechanisms for eligibility screening and administrative burden were found to hinder the CT recruitment process.^17^ Another study investigating clinical research associates’ views on barriers and facilitators to cancer CT recruitment also found system factors, such as increasing trial demands coupled with restrictive timelines, negatively impacted CT accrual.^16^

Understanding CT personnel's perspectives about what factors influence their decisions to offer a patient enrollment in a CT is important because their choices ultimately influence the overall success of CT accrual.^14^ Ethically, there is a need to balance the accrual demands of CTs with equitable access, as well as ensuring patient autonomy and informed consent.^18^, ^19^ This study explored, through an ethics lens, CT personnel's perspectives about patient recruitment to cancer CTs.

## 2 METHODS

This study was part of a larger qualitative study that explored patients’ CT decision‐making process, and the factors influencing patients’ autonomy and decisions to participate in cancer CTs. Qualitative methodologies have been identified as an important means to address CT accrual issues.^20^ A qualitative interpretive design in the current study permitted in‐depth exploration of a complex phenomenon by identifying themes and patterns from participants’ clinical experiences.^21^ Specifically the study aimed to: (a) identify CT personnel's views on the barriers and facilitators of patient autonomy within the trial recruitment process, and (b) examine justice and equity implications. Approval from the appropriate institutional research ethics board was received, and all participants provided informed consent before participating.

### 2.1 Recruitment

In 2011, CT personnel participants were recruited purposively at a large urban cancer center in Canada. Six nurses and six oncologists, involved in multiple aspects of cancer CTs (eg, patient recruitment, enrolment, and management or oversight of CTs), were recruited. Six participants identified as female and six as male. Participants were involved in trials of various phases related to breast, prostate, gynecological, gastrointestinal, head, and neck cancers. Participants were recruited until data saturation was reached.

### 2.2 Interviews

A researcher trained in bioethics theory and qualitative research (JAHB) conducted face‐to‐face interviews with CT personnel. Interviews lasted 30 minutes and were guided by an interview schedule developed by the research team. Open‐ended questions explored the challenges and facilitators participants perceived influenced patients’ decisions about CTs. Interviews also probed the specific factors and patient attributes that participants perceived to influence patients’ CT eligibility and an offer of enrollment.

### 2.3 Analysis

Interviews were digitally recorded and transcribed verbatim. Thematic and ethical analysis was applied to identify patterns in the data regarding how CT personnel presented trials to patients. An analytic template based on previous literature and the ethical theory of relational autonomy^22^ was applied to thematically organize the data according to the categories of personal, social, and structural influences. Linkages were explored between and among the identified themes to develop rich descriptions and interpretations.^23^

## 3 RESULTS

### 3.1 Gatekeeping

Participants discussed a process by which they navigated their ethical obligations within CT recruitment and determined which patients were most appropriate for a trial. They described needing to consider two overarching ethical commitments when offering enrollment in cancer CTs: ensuring patient autonomy through gaining informed consent to CTs and maintaining the scientific integrity of a trial by enrolling patients who would comply with the study protocol. However, participants identified tensions between these dual considerations. As a CT nurse explained: “My thought is the CT’s best interest or the patient's best interest too, but I also have to think about the CT because that's what my job is supposed to be.”

Participants described themselves as undertaking specific behaviors in response to perceived ethical obligations. They discussed enacting this behavior, which we identified as a form of gatekeeping, based on perceptions of patients’ *personal suitability* for a trial within the larger *structural context* (eg, healthcare system resources, trial protocol). These factors and contexts intersected to create an environment in which CT personnel determined which patients were most appropriate for a trial.

#### 3.1.1 Personal suitability

CT personnel discussed two kinds of eligibility prescreening they performed prior to extending a trial offer: pathology assessment and consideration of patients’ suitability for a trial. The assessment of pathology was relatively straightforward and dictated by the study protocol; however, assessing patients’ suitability for a trial was viewed as more challenging and subjective. An oncologist described this two‐pronged approach as follows:

Do they actually meet the eligibility? Do they have the right pathology ‐ the eligibility criteria? And that’s the easy part because that’s just a simple checklist. The next is probably the harder intangible stuff. Is this a person who really understands what a CT is? Is this a person who truly is suitable? Some trials have much more rigorous tests. Some are quite easy and simplistic in terms of the logistics. So, not every trial will suit every patient.

CT personnel reported approaching patients who they felt would be able to understand and fulfill all study requirements, thereby ensuring trial integrity and informed consent. Individual and social factors considered in establishing patient suitability for trials included competency, disease status, language and cultural background, geographic location, and level of family support.

CT personnel reported associating mental health with competency, and therefore, trial eligibility because patient capacity is often a criterion for trial inclusion. Patients with psychiatric disorders or addictions were thus frequently excluded from receiving a trial offer because they were perceived to lack mental capacity to provide fully informed consent. As one oncologist remarked:

Definitely the ones, you know, drug addiction, these are not patients who I put on trials. And patients with depression or psychiatric disorders I tend not to, either, (a) because of the medications and (b) they’re just – you don’t know whether they truly understand the trial and it can get very complicated.

Disease status and the emotional impact of cancer also influenced CT personnel's perceptions of patient suitability. Patients newly diagnosed with cancer, for example, were described as less suitable because many CT personnel believed these patients would be too overwhelmed to provide fully informed consent. Fear, anxiety, and the need for hope were also identified as emotions that potentially undermined patients’ capacity to understand and appreciate CT information.

Education and cultural background were also perceived to affect patients’ ability to provide informed consent. For instance, poor literacy and limited English language skills were flagged as potential barriers. CT personnel further believed the location of trials within urban cancer centers made CTs less suitable for rural and remote patients. Patients living outside major urban centers were perceived as less likely to have the resources to travel to and from the trial site. As one oncologist described:

You can tell a lot about whether it’s going to be a fit by incorporating where they live and what they do. I mean, obviously a logger who lives in [northern city], you can just guess he’s not going to have extended health benefits to bring him down so I look at things like that…So definitely geography, that’s a huge piece.

CT personnel were also aware that patients present with differing levels of social support, which could affect their ability to participate in a trial. They preferred to invite patients who had family nearby to help them understand trial information and provide logistical support (eg, driving to appointments). According to one oncologist:

Ideally, a married couple is always a good thing because a spouse is normally there for support. That’s not to say single people shouldn’t go on trials. Well‐supported single people – and even if they don’t have families but have a very supportive friend network…Where I tend to be a bit more worried is the person who lives on their own, doesn’t seem to have a lot of friends, may have a lot of pets.

Overall, CT personnel stated that the sort of person who is a good fit for a trial is an “exceptional person,” someone who is competent and able to think clearly about trial participation. It was not that some patients were inherently unsuitable, but comparatively, they would require extra resources to provide informed consent and ensure their continued participation in the trial, which neither the healthcare system nor trial could afford. Thus, CT personnel's decisions about whom to offer trial enrollment were described as being influenced by existing system capacity and available resources for CTs.

So even if I’m not thinking about the patient in front of me, I’m thinking, okay, is it a worthwhile investment of the extra time to offer this trial to this patient and in those sorts of cases, it isn’t, right? So, I can move on with my day much more quickly if I pre‐filter.

#### 3.1.2 Structural context

Personal factors impacting trial suitability intersected with structural considerations. Three structural factors were identified by CT personnel as influencing trial recruitment: CT complexity, the consent process, and time limitations in the healthcare system. These factors comprised the background conditions upon which CT personnel determined patients’ personal suitability for CTs.

Complex vs simple trial design was viewed by CT personnel to impact patients’ ability to understand what was involved in CT participation. Randomization, multiple study arms, frequent tests and procedures, and the inclusion of correlative research studies, contributed to perceived trial complexity. Trials with complex study protocols required CT personnel to limit recruitment to patients whom they believed would be able to comprehend study information.

Complex trials were also seen to strain limited clinical resources. A CT nurse explained how there were insufficient resources to support and coordinate “cumbersome trials” at all centers because sometimes “we can't make it work in our building ‐ if they [patient‐participant] need three CT scans a week, we can't do that here, because there's such a wait list for CT scans.” Inability to host trials at a cancer center immediately foreclosed the possibility of recruiting patients.

Consent forms were also described as problematic, causing CT personnel to decide who was most capable of comprehending the forms prior to offering and discussing a trial. Despite ethics requirements that consent forms should approximate a grade six reading level, CT nurses and oncologists perceived forms to be too detailed and complicated. Several oncologists believed the amount of information in consent forms was a hindrance to patients’ understanding and caused some patients to become “paralyzed,” unable to reach a decision. Understanding the consent form was perceived to be a greater challenge among patients without English as a first language, and those anxious or emotional as a result of their cancer diagnosis and treatment.

CT personnel expressed frustration regarding the limited time they had to discuss CTs with patients. Some participants perceived pressure to accrue patients within a specific time‐period or to obtain informed consent within predefined windows of enrolment (ie, before start of treatment). One nurse expressed exasperation with the current system and suggested CTs be redesigned to be patient centered:

I think that if we can give them as much time as possible, to not pressure them. But I don’t know how to fix that, because with some trials they are given the time, and with others you can’t because that is just the way the trial is built. So, then you have to go back even further and say, don’t write trials that are like this.

An oncologist suggested a triage system whereby eligible patients might attend a specialized clinic to discuss the CT being offered. In general, participants reflected on how CT infrastructure could be redesigned to better support patients’ understanding of CT information.

## 4 DISCUSSION

Our research highlights how lack of CT resources and CT personnel's gatekeeping judgments in response to ethical commitments may contribute to barriers to accrual notwithstanding trial exclusion criteria. Based on our findings, insufficient resources directed toward accrual activities forced CT personnel to prescreen patients sometimes more stringently than trial exclusion criteria or to interpret trial exclusion criteria (eg, mental competency) in a way that may be overly exclusionary. For example, persons with mental health issues and addictions were perceived by some CT personnel in this study to not have the capacity to understand a trial. This occurred despite research demonstrating considerable overlap in competence to consent among those with mental illness and the general population.^24^ Similarly, lack of family or other social support, living in a rural/remote location, and poverty could also negatively impact patients’ access to CTs due to assumptions CT personnel held about these patients’ ability to participate and lack of sufficient CT resources. The latter are well‐known barriers. Initiatives such as Eliminating Disparities in Clinical Trials (EDICT)^25^ and the National Cancer Institute's Community Clinical Oncology Research Program^26^ are underway to specifically target underserved populations in CTs through policy reform and collaborative research; for example, by linking community health practices with academic centers conducting cancer CTs. Our research contributes to an enhanced understanding of which populations may be understood as “underserved” within the confines of cancer CTs.

Gatekeepers to CTs exist at multiple levels of the research process, defined as those entities who “have the ability to allow or deny access to the resources required to support the conduct of clinical research.”^27^ Healthcare providers have been previously recognized as performing a gatekeeping role by allowing or denying patient access to research participation.^28^, ^29^ Gatekeeping protects vulnerable persons from harm and ensures other principles related to the ethical conduct of research are upheld. However, CT personnel's judgments may sometimes be based upon biases about the capacity of certain groups to understand CTs and take part. The result is that already marginalized groups, those who have mental health issues or addictions, lower socioeconomic status, or live in rural and remote areas, may have less or no access to CTs. Previous research had also found discussion about CTs and subsequent participation was associated with wealth and higher‐level education^30^ and patients with lower income had less chance of trial participation than higher income patients.^28^, ^31^ This is despite the fact that cancer incidence correlates with lower socioeconomic status.^32^ This inequity impacts not only the ability of these persons to access potentially helpful interventions but it also compromises the scientific integrity of CTs since interventions would not be appropriately randomized across all patient groups.

Lack of diversity within cancer CTs has been a longstanding issue and efforts to counteract it have been advanced by national organizations, such as the National Institutes of Health and the American Society of Clinical Oncology.^33^, ^34^ Despite valiant efforts, however, representation of racial and ethnic minorities in CTs has declined rather than increased over the past decade.^35^ Our study offers additional insights about how CT personnel's gatekeeping judgments and biases may contribute to barriers to the recruitment of diverse populations to cancer CTs. This understanding supports novel strategies to enhance equitable accrual, such as targeted education for CT personnel that addresses problematic biases and the need to develop creative solutions to enhance CT availability with limited resources.^36^

To address the structural issue of limited time and CT resources, a recent report identified the need for streamlined accrual strategies and trials designed to balance feasibility, accessibility, and scientific opportunity.^37^ For example, pragmatic CTs that compare effectiveness of treatment alternatives in real‐world settings and include representation from diverse patient populations have been one trial design proposed to simplify and improve the accrual process.^27^, ^38^

Streamlining written informed consent documents has also become a focus of global effort to ensure more meaningful and efficient consent procedures within trials.^39^, ^40^ Regulatory bodies in the United Kingdom recommend a layered approach to gaining informed consent, where potential CT participants review succinct, relevant, and truthful written trial information, with more detailed information available in the appendix or via online text.^39^ Revisions to the US Common Rule supporting the usage of more simplified consent forms are also currently underway.^40^

## 5 LIMITATIONS

Although data saturation was achieved, it is important to acknowledge that perceptions were described by a large proportion of a small yet highly engaged and knowledgeable sample of participants from a leading cancer care center. It is unknown how widespread such biases and gatekeeping judgments are in Canada or other countries, and the extent to which the perceived inability to offer CTs to certain patient populations may contribute to low accrual in cancer CTs. Participant observation of interactions between patients and CT personnel, as well as surveying a large sample of participants to examine the potential link between CT personnel biases, gatekeeping judgments, and accrual would provide further data. However, this study did uncover some biases that require attention. Although this research was conducted at a cancer center with a large CT program and within a country with universal health care, one institution may not reflect the standard of practice. Future research could investigate accrual issues using a larger CT personnel sample and in more diverse cancer CT settings.

## 6 CONCLUSION

Our study findings have broader ethical implications for justice and equitable access to CTs across different demographic groups. Specifically, gatekeeping judgments based on biases instead of sound evidence may negatively influence equitable access to CTs, especially for populations marginalized by factors such as geography, poverty, mental health/addiction, immigration status, and language. These factors impact the ability of persons to exercise their autonomy, access potentially helpful interventions, and contribute to scientific knowledge and advancements in care. Enhanced CT processes, resources, education, and tools that address biases and support patient CT decision‐making may help ensure diverse recruitment to trials, which will allow for advancements in individualized approaches to cancer control while also promoting health equity.

## CONFLICT OF INTEREST

The authors have no conflicts of interest to report.

## AUTHOR CONTRIBUTIONS

JAHB: conceptualization, data curation, analysis, methodology, project administration, writing—original draft, and writing—review and editing. MTK: analysis; writing—original draft, and writing—review and editing. KG: analysis; writing—original draft, and writing—review and editing. KC: analysis; writing—original draft, and writing—review and editing. AH: analysis; writing—original draft, and writing—review and editing. PR: analysis; writing—original draft, and writing—review and editing. LGB: conceptualization; analysis; writing—original draft, and writing—review and editing.

## Data Availability

Data available on request from the authors.
